# Sequential Use of Tezepelumab and Bronchoscopic Lung Volume Reduction With Endobronchial Valves in a Patient With Severe Asthma‐COPD Overlap and Heterogeneous Emphysema: A Case Report

**DOI:** 10.1002/rcr2.70407

**Published:** 2025-11-12

**Authors:** Jonas Herth, Daniel Franzen

**Affiliations:** ^1^ Department of Internal Medicine Hospital of Uster Zurich Switzerland; ^2^ Department of Internal Medicine University Hospital Zurich Zurich Switzerland; ^3^ Department of Pulmonology Hospital of Uster Zurich Switzerland; ^4^ Faculty of Medicine University of Zurich Zurich Switzerland

**Keywords:** asthma‐COPD overlap, bronchoscopic lung volume reduction, emphysema, endobronchial valves, Tezepelumab

## Abstract

Asthma‐COPD overlap (ACO) carries greater symptom burden, frequent exacerbations and impaired quality of life compared with asthma or COPD alone. Evidence‐based advanced therapies are lacking, and management is typically extrapolated from existing guidelines. Tezepelumab, an anti‐thymic stromal lymphopoietin antibody, reduces exacerbations and improves lung function in severe asthma. Bronchoscopic lung volume reduction (BLVR) with endobronchial valves benefits selected patients with advanced emphysema and hyperinflation despite optimal therapy. A 71‐year‐old woman with severe ACO, frequent exacerbations and hyperinflation despite triple inhaled therapy was treated sequentially with tezepelumab and BLVR. Tezepelumab improved airway control and reduced exacerbations; BLVR subsequently addressed persistent hyperinflation. Over 2 years, the patient achieved sustained improvements in lung function, St. George's Respiratory Questionnaire score and annual exacerbation rate. This case highlights the potential benefit of a combined anti‐inflammatory and interventional approach in difficult‐to‐treat ACO, a population for whom evidence‐based advanced therapies remain limited.

## Graphical abstract

Sequential treatment with tezepelumab followed by bronchoscopic lung volume reduction achieved sustained improvements in lung function, symptoms and exacerbation frequency in a patient with severe asthma‐COPD overlap.

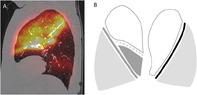

## 1 Introduction

Asthma‐COPD overlap (ACO) represents a complex and challenging phenotype characterised by persistent airflow limitation and features of both asthma and chronic obstructive pulmonary disease (COPD), often resulting in a greater symptom burden, more frequent exacerbations and reduced quality of life compared to either disease alone. Management usually combines pharmacologic and non‐pharmacologic measures, but a subset of patients remains symptomatic or experiences frequent exacerbations, highlighting the need for novel therapeutic approaches. Evidence‐based recommendations for advanced therapies in ACO are lacking, and management is typically extrapolated from asthma and COPD guidelines [1].

In severe asthma, Tezepelumab, a monoclonal antibody targeting thymic stromal lymphopoietin (TSLP) reduces exacerbations and improves lung function and quality of life across phenotypes, including those with low eosinophil counts or nonallergic disease [2]. For patients with advanced emphysema and hyperinflation, bronchoscopic lung volume reduction (BLVR) with endobronchial valves can improve symptoms and outcomes when careful imaging‐based selection is applied [3]. Combining biologic therapies with interventional procedures to address both inflammatory and persistent hyperinflation may provide a comprehensive approach to managing patients with difficult‐to‐treat ACO. We report a patient with severe ACO and persistent symptoms despite triple inhaled therapy, who experienced significant clinical improvement following sequential treatment with Tezepelumab and BLVR.

## 2 Case Report

A 71‐year‐old woman, ex‐smoker (40 pack‐years), was diagnosed with ACO with a predominant COPD component classified as Global Initiative for Chronic Obstructive Lung Disease Stage 3. Her history included allergic rhinitis, with onset in early adulthood, characterised by sensitisation to dust mites and birch pollen, and seasonal rhinoconjunctivitis. Despite maximal inhaled therapy, corresponding to an inhaled corticosteroid dose of approximately 700 μg budesonide equivalent per day, she experienced four exacerbations annually and progressive exertional dyspnea severely limiting her quality of life.

**FIGURE 1:**
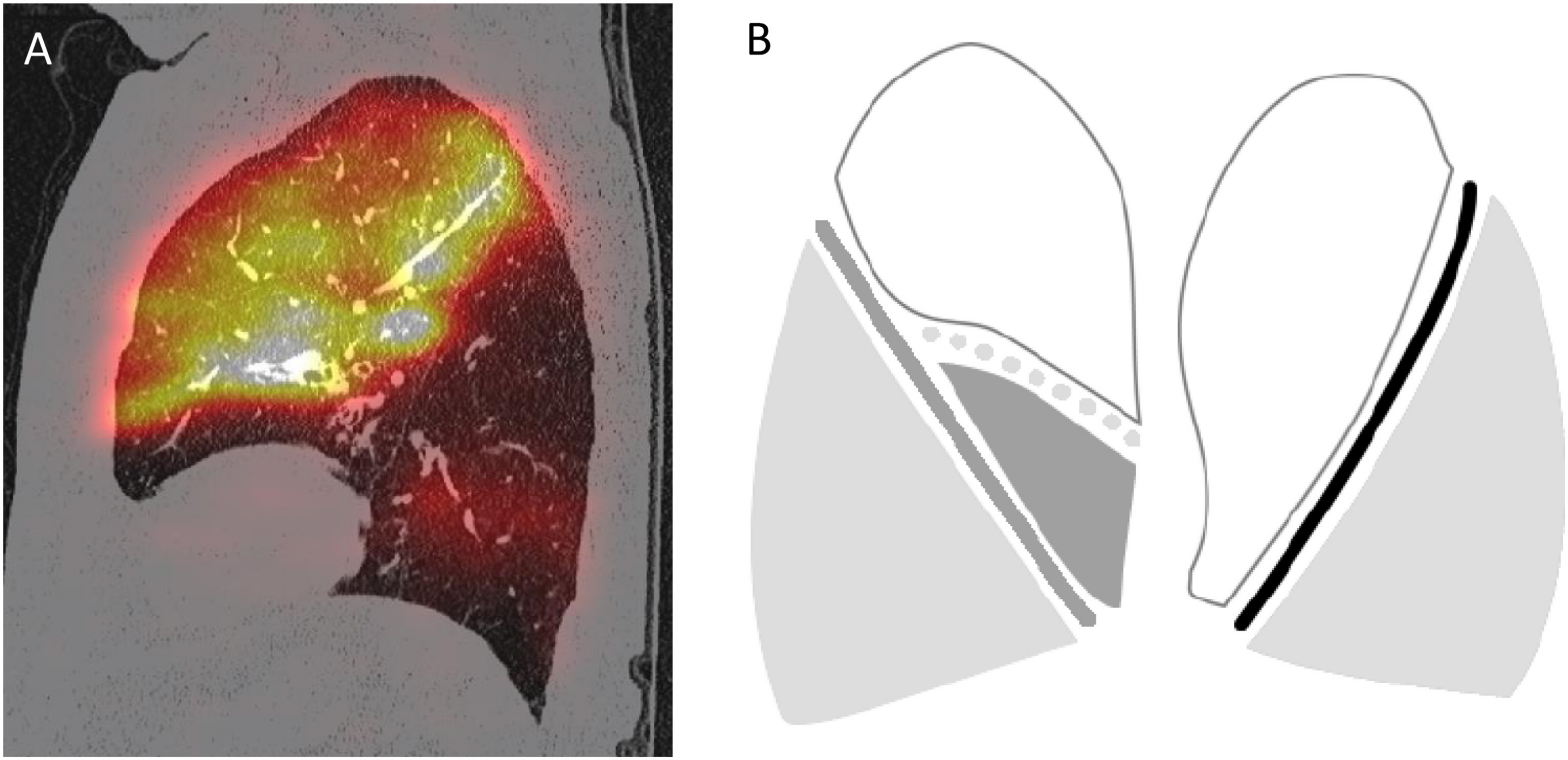
(A) Quantitative lung analysis performed using StratX Lung Analysis Platform (Pulmonx Corporation, Redwood City, CA, USA). (B) Sagittal view of lung perfusion scintigraphy obtained using single‐photon emission computed tomography (SPECT) (OptimaTM CT540, GE Healthcare, Chicago, IL, USA).

Spirometry revealed severe, non‐reversible airflow obstruction with a post‐bronchodilator forced expiratory volume in the first second (FEV1) of 0.81 L (39% predicted). Body plethysmography confirmed hyperinflation (Table 1). Lung perfusion scintigraphy and single photon emission computed tomography/computed tomography (SPECT/CT) demonstrated markedly reduced perfusion of the left lower lobe, accounting for 5% of total tracer counts (Figure 1A). Quantitative analysis with StratX revealed complete interlobar fissures, with 55% of voxels in the left lower lobe exhibiting a density less than 910 Hounsfield units (Figure 1B), lobe volume was 683 mL (StratX) and 669 mL (SPECT). Although BLVR was feasible, frequent exacerbations posed procedural risk. Given severe allergic asthma with recurrent exacerbations, low eosinophils (0.1 G/L), low IgE (33.1 kU/L), allergen sensitisation and no long‐term corticosteroids, the patient qualified for Tezepelumab. Tezepelumab was chosen over Dupilumab, as it is indicated for severe asthma with frequent exacerbations regardless of eosinophil or IgE levels. After two doses, exertional dyspnea and overall condition improved significantly. 3 months later, BLVR with six endobronchial valves was performed in the left lower lobe without complications. Over 2 years of follow‐up, the patient showed great benefit in most aspects, with St. George's Respiratory Questionnaire (SGRQ) score showing marked quality‐of‐life improvement and exacerbations declining from four to two per year (Figure 1).

**TABLE 1:** Pulmonary function before and after Tezepelumab treatment (11/2022) and BLVR with endobronchial valves (02/2023).

Time	FVC, litres (%predicted)	FEV1, litres (%predicted)	FEV1/FVC, %	TLC, litres (%predicted)	RV, litres (%predicted)	RV/TLC, %	TLCO (%predicted)	6‐MWD, metres	SGRQ	CAT	mMRC	FeNO, ppb	Eos, kU/L	IgE, G/L
Pre‐ Tezepelumab (1.5 years)	1.80 (67)	0.87 (42)	48	5.86 (128)	4.05 (210)	69						14	0.2	38
Pre‐ Tezepelumab (6 month)	1.46 (56)	0.73 (36)	49	5.46 (121)	3.91 (203)	71	3.83 (58)					16	0.3	
Pre‐ Tezepelumab (1 month)	1.70 (65)	0.81 (40)	47	6.09 (135)	3.72 (193)	61	4.79 (72)	446	80	36	4	18	0.1	33.1
4‐weeks post BLVR	1.65 (64)	0.92 (46)	56	5.02 (111)	2.93 (151)	58	3.90 (59)	477	58	31	3			
12‐month post BLVR	2.00 (80)	1.06 (55)	53	5.18 (118)	2.95 (153)	56	4.20 (67)	490	17	11	1	16	0.1	
24‐month post BLVR	2.22 (92)	0.96 (51)	43	4.61 (107)	2.21 (115)	48	4.20 (67)	405				8	0.5	

Abbreviations: 6‐MWD, 6‐minute walking distance; BVRS, bronchoscopic lung volume reduction surgery; CAT, COPD Assessment Test; Eos, Eosinophiles; FeNO, Fractional Exhaled Nitric Oxide; FEV1, forced expiratory volume in 1 second; FVC, forced vital capacity; mMRC, Modified Medical Research Council; RV, residual volume; SGRQ, St. George's Respiratory Questionnaire; TLC, total lung capacity; TLCO, transfer factor of the lung for carbon monoxide.

## 3 Discussion

ACO is a heterogeneous clinical entity associated with greater symptom burden, more frequent exacerbations and reduced quality of life compared to asthma or COPD alone. The absence of a consensus definition and the exclusion of ACO patients from most clinical trials have limited evidence‐based recommendations beyond first‐line inhaled therapies [1]. This case demonstrated the potential benefit of combined anti‐inflammatory and bronchoscopic intervention in a patient with difficult‐to‐treat ACO. The risk of post‐procedural complications in patients with frequent exacerbations highlighted the importance of stabilising airway inflammation before intervention [3]. Tezepelumab was selected as initial therapy due to its demonstrated efficacy in patients with severe asthma. In pooled analyses of the PATHWAY and NAVIGATOR trials, it reduced annualised exacerbation rates by about 60% and improved lung function and patient‐reported outcomes [2]. Data from the COURSE trial suggest possible benefit in COPD patients with higher blood eosinophils, although the primary endpoint was not met and further studies are required to clarify its role [4]. By addressing airway inflammation with Tezepelumab, the reduction in exacerbations facilitated safe and effective BLVR.

BLVR with endobronchial valves is an established intervention for selected patients with severe emphysema and hyperinflation, resulting in improvements in lung function, exercise capacity and quality of life. As the procedure carries risks such as pneumothorax and post‐procedural infection, careful patient selection and monitoring in experienced centers are essential. Optimal outcomes are achieved in patients with heterogeneous emphysema, intact fissures and absence of collateral ventilation [3]. The sustained FEV_1_ increase (12%, 0.16 L) over 2 years aligns with improvements reported for Tezepelumab in severe asthma and BLVR in advanced emphysema. The reduction in SGRQ score (63 points) after 2 years far exceeds the minimal clinically important difference, indicating a profound improvement in health‐related quality of life. Both Tezepelumab and BLVR have independently been linked to significant improvements in patient‐reported outcomes, and the reduction in annual exacerbations from four to two per year is consistent with the 40%–60% reduction observed with Tezepelumab in severe asthma [2, 5, 6]. These findings support the idea that targeting both, airway inflammation with Tezepelumab and structural lung damage with BLVR can yield complementary and durable benefits in selected patients with severe ACO.

Limitations include the single‐patient design, limiting generalisability and the short interval between therapies, which prevents clear attribution of individual effects. High cost and limited availability may further restrict broader application.

In summary, this case demonstrates the potential of a combined therapeutic strategy targeting airway inflammation and structural lung dysfunction in difficult‐to‐treat ACO. Although current evidence remains limited, the observed clinical benefit highlights the need for further investigation of integrated biologic–procedural approaches in ACO.

## Author Contributions

Jonas Herth wrote the original draft of the manuscript. Daniel Franzen performed the bronchoscopy intervention. All authors reviewed and approved the final draft of the manuscript for publication.

## Consent

The authors declare that written informed consent was obtained for the publication of this manuscript and accompanying images using the consent form provided by the Journal.

## Conflicts of Interest

Daniel Franzen reports receiving speaker honoraria from AstraZeneca, Pulmonx, GlaxoSmithKline (GSK), OM Pharma and Sanofi Aventis. Jonas Herth has no conflicts of interest to declare.

## Data Availability

The data that support the findings of this study are available from the corresponding author upon reasonable request.
